# The Functional Molecular Biopsy: Exercise-Induced Transpulmonary Multi-Omic Profiling in Pulmonary Arterial Hypertension

**DOI:** 10.3390/ijms27209130

**Published:** 2026-10-15

**Authors:** Fernanda P. Oliveira, Thais C. F. Menezes, Claudia Mickael, Rudolf K. F. Oliveira

**Affiliations:** 1 Division of Respiratory Diseases, Department of Medicine, Hospital São Paulo, Federal University of São Paulo (UNIFESP), São Paulo 04024-002, SP, Brazil; fernanda.paz@unifesp.br (F.P.O.);; 2 Division of Allergy, Pulmonary, and Critical Care Medicine, Department of Medicine, University of Colorado Denver, Anschutz Medical Campus, Aurora, CO 80045, USA

**Keywords:** pulmonary vascular disease, exercise right heart catheterization, transpulmonary concentration gradient, proteomics, metabolomics, exercise hemodynamics

## Abstract

Pulmonary hypertension (PH) is a disease in which diverse pathogenic drivers converge to promote inflammatory vascular remodeling, ultimately leading to right ventricular (RV) dysfunction and heart failure. Exercise right heart catheterization (RHC) reveals abnormal pulmonary vascular responses early in the disease course and improves diagnosis and risk stratification in PH. Despite major advances in PH research, direct access to the biological processes driving pulmonary vascular remodeling remains limited. Existing clinical assessments predominantly capture the consequences of disease rather than its causes, while tissue-based studies are constrained by limited access to early-stage human samples and the inability to perform serial measurements. This creates a critical need for methodologies capable of interrogating pulmonary vascular biology *in vivo* and across the disease continuum. We propose that combining exercise RHC with paired transpulmonary omics measurements may provide such an opportunity. The strategy of measuring the change in molecular concentrations across the pulmonary circulation during physiological stress has the potential to function as a dynamic molecular biopsy of the pulmonary vasculature, linking abnormal exercise hemodynamics to the biological pathways that drive disease, although current supporting evidence remains limited to small, heterogeneous proof-of-concept cohorts. In this review, we examine the physiological basis, technical framework, biological insights, and translational implications of this emerging approach for PH.

## 1. Introduction

Pulmonary hypertension (PH) is a heterogeneous and multifactorial disease characterized by progressive pulmonary vascular remodeling, increased pulmonary vascular resistance, and eventual right ventricular failure. One year mortality exceeds 20% in the high-risk group of patients with pulmonary arterial hypertension (PAH) [1]. The consequent RV dysfunction and hypoxia have implications for multiple organ systems including the autonomic nervous system, kidneys, brain, immune system [2], and skeletal muscle [3], through inter-organ crosstalk mediated by circulating pro-inflammatory cytokines.

A fundamental challenge in managing PH is its pathobiological diversity. The disease is clinically divided into five groups: pulmonary arterial hypertension (PAH), pulmonary hypertension associated with left heart disease, PH associated with chronic lung disease and hypoxia, chronic thromboembolic pulmonary hypertension, and PH due to unclear/multifactorial mechanisms [1]. This classification is based on underlying pathophysiological mechanisms, and each group shows distinct histological features, remodeling patterns and responses to therapy [4]. For instance, in PAH, risk factors include shear stress, epigenetic factors, female sex and genetic mutations in BMPR2, a gene encoding a TGF-β type II receptor [5,6].

Currently available clinical tools, including traditional resting hemodynamics and protein biomarkers like natriuretic peptides (e.g., BNP and pro-BNP) do not reliably distinguish between different clinical types of PH and mostly reflect cardiac dysfunction rather than the underlying pulmonary vascular pathology [7]. They fail to capture the active, local, upstream biological drivers of pulmonary vascular remodeling such as endothelial dysfunction and perivascular inflammation.

There remains a gap in therapeutic strategies targeting vascular remodeling and inflammation [8], highlighting the need for more etiology/stage-specific data. This narrative review examines whether exercise-enhanced transpulmonary molecular sampling can bridge this gap. This narrative synthesis draws on a targeted PubMed/MEDLINE search of pulmonary hypertension, exercise hemodynamics, and transpulmonary omics literature from April to June 2026 and last updated on 1 October 2026. The principal search combination was “pulmonary hypertension”, “exercise”, “exercise right heart catheterization” “transpulmonary gradients”, “proteomics”, “metabolomics” and “pulmonary circulation”, supplemented by additional sources selected for their direct relevance to the conceptual framework proposed here. No date restriction on publication was applied.

## 2. The Limits of Resting Assessment

PH is diagnosed by a resting mean pulmonary artery pressure (mPAP) > 20 mmHg at on right heart catheterization (RHC); the pre-capillary phenotype relevant to this review additionally requires a pulmonary vascular resistance (PVR) > 2 Wood units and a pulmonary arterial wedge pressure (PAWP) ≤ 15 mmHg [1]. However, resting hemodynamics provides a static snapshot and may fail to detect early endothelial dysfunction because vascular distensibility is not challenged under steady-state conditions, which is an early abnormality in PH [9]. In addition, it may not reveal a latent or early-stage inability to augment RV cardiac output in response to the increased afterload induced by physical stress [10].

Existing molecular tools are similarly inadequate for isolating local pulmonary vascular pathology. In current practice, BNP and NT-proBNP are the most widely used protein biomarkers in PAH for diagnosis and risk stratification, with low-risk thresholds of BNP < 50 pg/mL and NT-proBNP < 300 pg/mL associated with lower 1-year mortality risk [11]. Other biomarkers including creatinine [12], STC1 and IL6 [13,14] have also been associated with outcomes in idiopathic PAH, and inflammatory markers are shared across PH etiologies [15]. While the use of proteomic clusters has been proposed to improve accuracy by capturing different disease pathways simultaneously, establishing specificity for the intrinsic pulmonary vascular processes remains a challenge, and there remain gaps in the pathophysiology protein pathways [16]. Also, these peripheral signals dilute and mask the localized, low-abundance biological processes occurring within the pulmonary microenvironment.

Another major research challenge is the restricted access to early-stage or untreated human tissue, as premortem histopathology is rarely performed [17] and typically represents end-stage disease, possibly with repair and compensatory mechanisms combined. Animal models such as sheep, dogs, pigs and horses are a controversial alternative when studying PH due to different metabolic, anatomical and measurement technique differences [18,19]. Similarly, rodent models used for mechanistic studies of vascular disease and preclinical drug testing, such as the Sugen-hypoxia, chronic hypoxia and *Schistosoma mansoni* murine model fail to recapitulate many features of human PH, such as the severe angioproliferation, RV failure, and disease progression [20,21,22,23].

Furthermore, ex vivo platforms fail to replicate the complex, living vascular environment. Precision-cut lung slices (PCLS) have shown preserved vasoreactivity in human lung tissue, but one of its limitations is inconsistencies across research groups in preparation and culture conditions [24]. Moreover, the samples come from lung biopsies of patients with other lung pathologies or from explanted tissue, which generally reflects advanced disease [25,26], and endothelium survival in culture is short [27]. Together, these limitations create a critical knowledge gap; we need a window into pulmonary vascular biology that is (a) non-destructive, (b) accessible across disease stages, (c) lung-specific rather than systemic, and (d) sensitive to early, pre-clinical dysfunction. Table 1 summarizes current available tools for PH assessment.

## 3. Exercise as a Physiological Probe of Pulmonary Vascular Reserve

Healthy pulmonary circulation is a low-pressure, high-flow circuit that must adapt during exercise to accommodate the rise in cardiac output (CO), mean pulmonary artery pressure (mPAP), pulmonary arterial wedge pressure (PAWP) and RV afterload. In normal physiology, as CO increases, PVR decreases slightly, while the RV increases contractility, heart rate and diastolic function to maintain blood flow through a compliant vascular bed. This unique hemodynamic adaptation relies on vascular distensibility reserve—the recruitment and distension of previously unperfused or under-perfused pulmonary capillaries. However, sustained exposure to increased afterload may ultimately lead to RV hypertrophy and dilatation [18,28,29,30].

**Figure 1:**
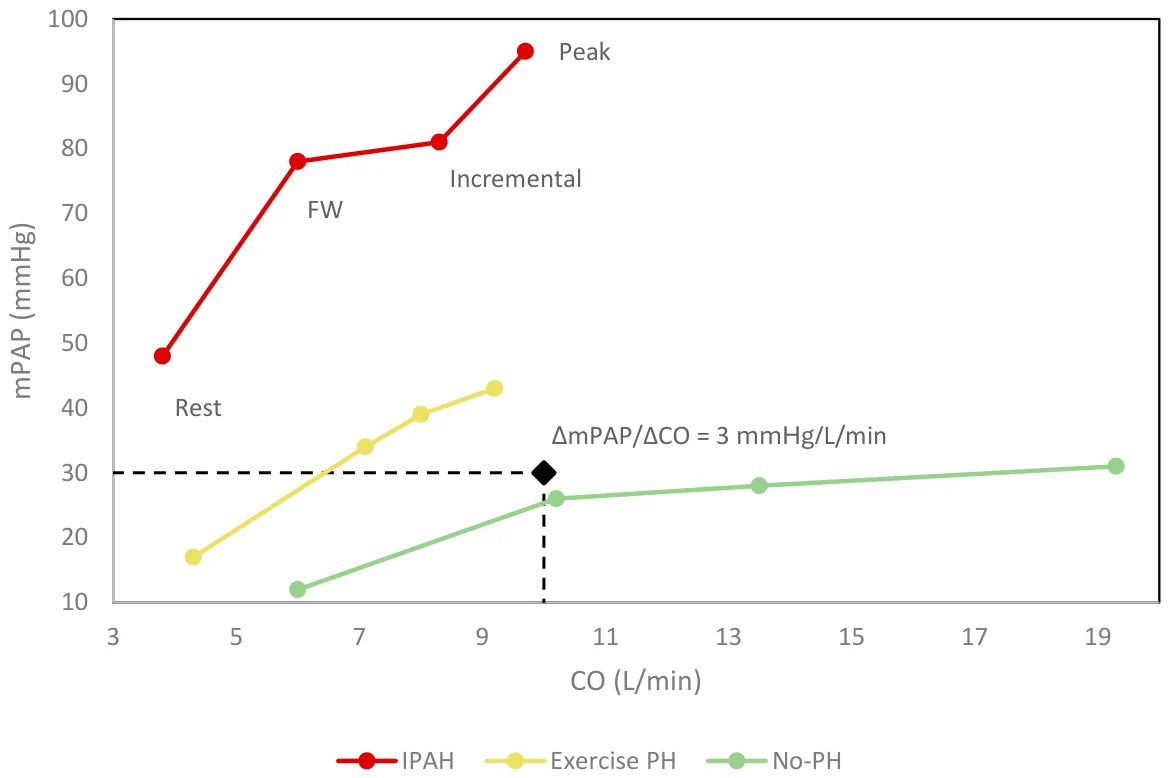
Multipoint pressure–flow relationship during exercise. The graph illustrates a didactic and representative schematic data on hemodynamic response to exercise in four points: rest, free-wheel, incremental and peak exercise in distinct diagnostic groups: idiopathic pulmonary arterial hypertension (IPAH, red), exercise-induced pulmonary hypertension (Exercise PH, yellow), non-pulmonary hypertension (No-PH, green). Exceeding the slope threshold (ΔmPAP/ΔCO) alone does not establish a precapillary cause; pulmonary artery wedge pressure is needed for interpretation. mPAP: mean pulmonary artery pressure. CO: cardiac output.

Early structural remodeling causes loss of vascular distensibility, preventing capillary recruitment during exercise. Consequently, the pulmonary vascular bed cannot accommodate increased blood flow; PVR fails to fall, and mPAP rises disproportionately relative to CO, resulting in a pathological mPAP/CO slope greater than 3 mmHg⋅min⋅L^−1^ during exercise right heart catheterization [1,31]. This abrupt afterload mismatch rapidly accelerates RV–PA uncoupling. The RV is forced to operate at a markedly elevated right ventricular stroke work index (RVSWi), which, combined with reduced pulmonary vascular compliance, limits peak oxygen consumption (peak VO_2_) and triggers exertional dyspnea—an early clinical manifestation of occult RV failure [32,33,34]. Exercise RHC unmasks these hemodynamic and mechanical abnormalities before they manifest under resting, steady-state conditions [35]. Figure 1 exemplifies flow and pressure response to exercise in distinct groups.

Reliable quantification of the mPAP/CO slope depends on accurate peak-exercise cardiac output measurement. Thermodilution remains the practical standard at most centers, given the added complexity of direct Fick (simultaneous arterial and mixed venous blood gas sampling with co-oximetry, a metabolic cart, and measured VO_2_). While thermodilution performs reasonably well for the purpose of PH classification, its agreement with direct Fick narrows at peak exercise [36,37]. Consequently, when assumed VO_2_ or thermodilution-derived CO is used in the underlying studies discussed below, it represents a potential source of measurement uncertainty that should be considered when interpreting reported gradients. Exertional abnormalities define a critical pre-clinical window. Exercise hemodynamics can also differentiate ambiguous phenotypes, such as unmasking left heart disease (Group 2 PH), which carries important therapeutic implications because PAH-targeted therapy can be deleterious in this group [38]. Furthermore, abnormal exercise responses have been described in individuals at risk of developing PH, such as family members of patients with hereditary PH [39], and are also associated with increased mortality in systemic sclerosis prior to the development of PH, reinforcing their prognostic relevance [40].

In PH patients, dysfunctional inflammatory signaling is a key pathological feature [15,41]. Beyond uncovering macrovascular dysfunction, exercise acts as an intense local provocative stimulus that actively amplifies underlying pathological molecular pathways. In healthy individuals, it triggers an acute stress response, amplifying CD8 T cells, macrophage and natural killer cell activity, as well as serum immunoglobulins and cytokines such as TNF-α [42,43]. In the presence of disease pathology, this mechanical stress deregulates core vasoactive and remodeling cascades, including sympathetic nervous system downregulation of β-adrenoreceptors in the RV myocardium, nitric oxide, prostacyclin, thromboxane, endothelin-1, and vascular endothelial growth factor (VEGF) signaling [44,45], while activating regional alveolar hypoxia pathways driven by hypoxia-inducible factors (HIF) [46]. Simultaneously, the combination of high physical pressure and pre-existing endothelial injury causes loss of the endothelial barrier function, widening cellular tight junctions and markedly increasing microvascular permeability [47] and exacerbating RV–PA coupling impairment [48]. By driving the active cellular extrusion and leakage of localized cellular contents into the passing bloodstream, we hypothesize that exercise increases the release of locally produced molecules into the passing bloodstream, concentrating local pathological markers that are otherwise diluted at rest.

Because exercise both unmasks hemodynamic abnormality and amplifies the biological signals driving that abnormality, it offers a physiological window, not merely for hemodynamic characterization, but for real-time molecular phenotyping of pulmonary vascular disease. This physiological window remains underused in translational PH research; realizing its diagnostic and mechanistic potential requires a systematic framework for capturing and interpreting these exercise-induced molecular signals, which the following section describes.

## 4. Functional Molecular Biopsy: Principle and Technical Framework

Simultaneous blood sampling from distinct vascular sites provides concentration gradients, estimating secretion and absorption of substrates across a specific organ. This is an established principle in cardiovascular studies, used to calculate myocardial and systemic metabolic kinetics in complex disease states [49,50].

**Figure 2:**
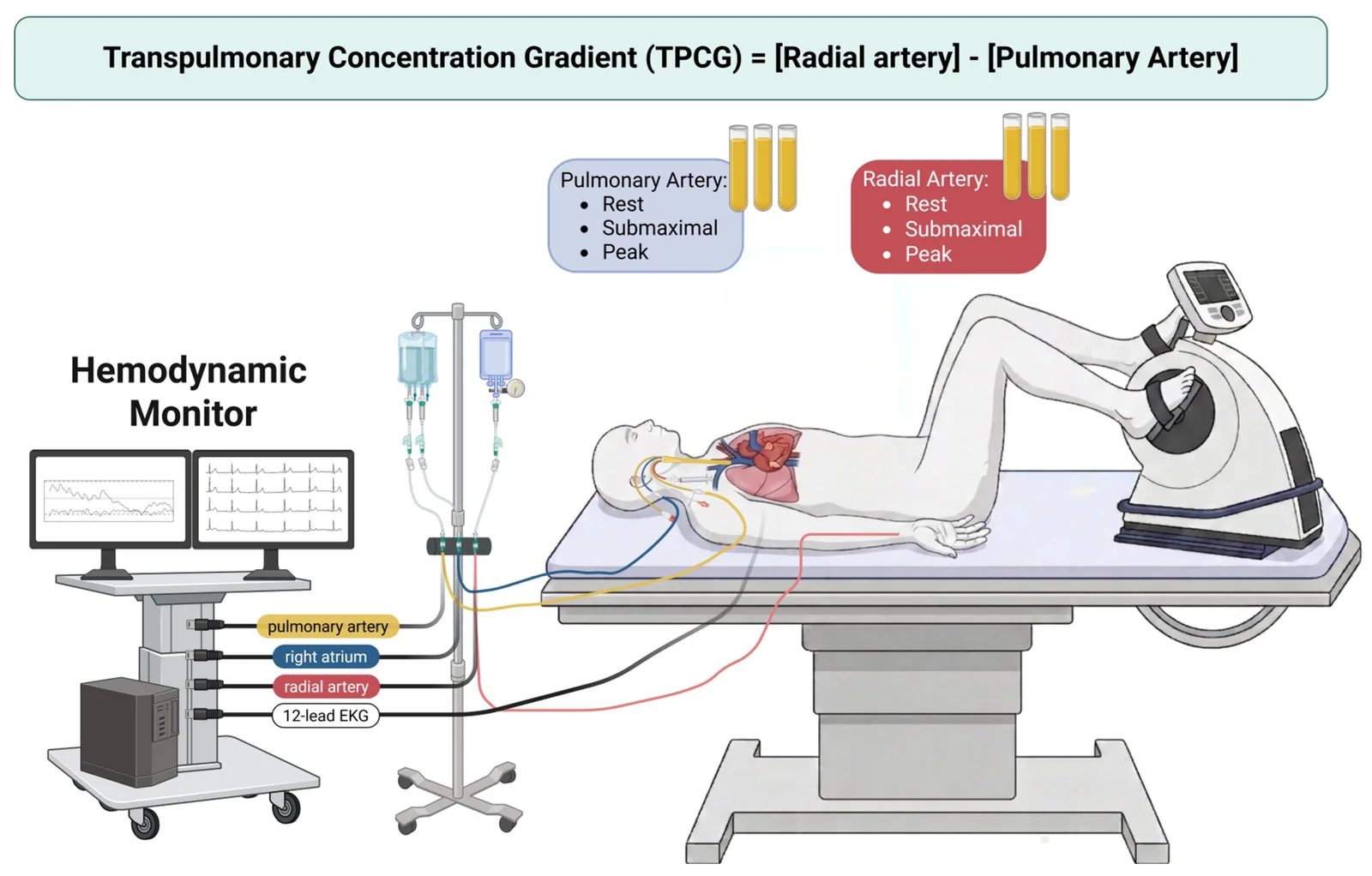
Schematic workflow of blood sampling during exercise right heart catheterization. Paired blood samples are collected from pulmonary artery (via Swan–Ganz catheter) and radial artery (via peripheral line) during the exam in different stages of exercise to determine the transpulmonary concentration gradient (TPCG), defined as the difference in concentration between radial and pulmonary artery plasma markers. Created in BioRender. Menezes, T. (2026) https://BioRender.com/mv862y5.

When applied to the pulmonary circulation, collecting plasma directly from the pulmonary artery (representing mixed venous inflow) and comparing it with a synchronous systemic arterial sample (representing pulmonary venous outflow) enables calculation of transpulmonary concentration gradients (Figure 2). This gradient may provide insight into net pulmonary secretion and uptake, although it cannot precisely define which compartment within the pulmonary system (e.g., vessels, parenchyma); also there is a minor addition of venous blood from drainage of bronchial veins and thebesian veins into the left atrium and ventricle, but it only corresponds approximately to 1–2% of the blood in systemic arterial circulation [51,52]. Molecules showing a positive transpulmonary gradient (where systemic arterial concentration exceeds pulmonary artery concentration) are being actively released or shed by the lung tissue. Conversely, molecules showing a negative gradient are being cleared or extracted by the pulmonary circulation. This approach offers an opportunity to reveal localized dysregulation across different PH etiologies and stages [53].

Proteins are classified by spatial distribution into circulating, locally secreted and intracellular pools. Although most proteins remain intracellular, a subset of about 730 enters the circulation, and many stay locally active after secretion [54]. They represent the final pathway of complex biological processes such as immune activation, and as immune activation varies across PH subtypes, including hypoxia-driven disease, infection-associated disease, and connective tissue disease-associated disease, proteomic analysis may capture subtype-specific molecular signatures that reflect distinct pathogenic mechanisms [55].

At rest, isolating these local gradients remains a major technical challenge. While proteomics from lung tissue [41], endothelial cells from transplanted lungs [56], and peripheral muscle [57] can provide mechanistic insight, plasma in addition offers clinical accessibility, serial sampling and lower risk than biopsy, which may also not be a representative sample of the organ’s pathology [16,58]. Furthermore, plasma contains proteins across a wide range of sizes, glycosylated forms and dynamic concentration in tissues. For example, albumin is a large protein with a plasma concentration of 50 mg/mL (55% of total protein) while troponin, a validated prognostic biomarker for PH, is present in levels of 10–20 ng/L and cytokines such as IL-6 are even less abundant, at concentrations around 5 pg/mL [59]. At rest, early or localized disease signaling from the pulmonary vasculature is easily diluted within the systemic circulation, often falling below the lower limit of detection. The introduction of exercise right heart catheterization acts as a provocative stimulus. The exercise-induced surge in pulmonary blood flow and localized endothelial shear stress can amplify active cellular secretion and local barrier shedding, depending on change in release, uptake and flow, and low-abundance markers at rest may have their detection increased within the transpulmonary transit with exercise stress.

*Proposed practices and technical requirements for interpreting a small paired difference:* Transpulmonary gradient measurement reliability depends on several design elements: (a) *Synchronization:* pulmonary arterial and arterial samples should be drawn simultaneously, because transit time through the lung shortens as cardiac output rises and a drifting workload decouples inflow from outflow. Cardiac output at each stage (thermodilution or Fick; [36,37]) should be recorded for the same interval. (b) *Processing:* paired samples should use the same tube type, the same discarded dead-space volume, identical time to centrifugation and storage, and be run on the same plate or batch, so that processing does not generate a spurious gradient [60,61]. (c) *Hemolysis and plasma-volume shifts:* samples should be screened for hemolysis, which releases intracellular proteins and metabolites, and exercise-related hemoconcentration should be addressed by reporting hematocrit or hemoglobin at each site and, where relevant, correcting concentrations accordingly. (d) *Analytical error and detection limits:* We propose a resting paired-sampling reference to define the null-gradient range [60]. (e) *Absolute versus relative measures:* assays reporting absolute concentrations (e.g., ELISA; [61]) allow a concentration difference and, combined with plasma flow (cardiac output × [1 − hematocrit]), a net mass flux across the lungs. Relative platform signals such as Olink NPX are log2-scaled and have no absolute unit; their difference is a log fold change and cannot be converted directly into a concentration difference or mass flux [60,62]. Findings from such platforms should be reported as relative gradients, and key candidates confirmed with an absolute assay.

Several high-throughput proteomic platforms can be applied to transpulmonary gradient analysis, each with different tradeoffs. Untargeted mass spectrometry (LC–MS) offers unbiased, discovery-scale coverage but lower throughput [63,64]. Olink’s proximity extension assay offers pre-specified, disease-relevant panels [62]; and SomaScan’s aptamer-based platform offers the broadest coverage at generally lower per-protein precision [65]. A critical methodological caveat for any gradient study spanning platforms or seeking external validation is that inter-platform concordance between Olink and SomaScan is modest and highly protein-dependent: large head-to-head comparisons report median inter-platform correlations, with only a minority of shared proteins reaching high correlations [66,67]. Therefore, a candidate biomarker identified as a transpulmonary gradient on one platform may not be replicated on another, and cross-platform validation should be planned next. Standardization of the exercise protocol itself is an equally unresolved variable. Ramp versus incremental loading and seated versus supine cycling produce different hemodynamic trajectories [35] and, plausibly, different gradient dynamics, though this has not yet been systematically studied for transpulmonary omics sampling specifically.

The clinical feasibility of this functional molecular biopsy is supported by early data, albeit from small and heterogeneous cohorts. Initial studies in metabolomics are combined with exercise isolated localized metabolite expression patterns in the pulmonary artery [68] and in the superior vena cava, as well as the pulmonary artery and ascending aorta at rest during active catheterization [69]. More recently, in pulmonary vascular disease associated with HFrEF, a study combining rest RHC with paired transpulmonary proteomic sampling (wedged vs. unwedged pulmonary catheter positions) reported molecular detection patterns consistent with local microvascular stress and right-ventricular matching that were not evident in peripheral, single-site sampling [60]. This provides evidence that exercise-based transpulmonary proteomic sampling captures the real microvascular and RV–PA signal in a cardiopulmonary population, though in Group 2 PH-HFrEF rather than PAH.

With this technical framework established, the following section examines what biological information these gradients are capable of revealing and what it might mean for understanding the disease mechanisms.

## 5. Biological Insights Revealed by Exercise-Induced Transpulmonary Gradients

Throughout this section, we distinguish analytes with existing transpulmonary gradient evidence from candidates nominated by tissue and systemic biology but not yet tested with this method, the latter representing hypotheses for future gradient-based validation rather than established findings.

### 5.1. Endothelial Dysfunction and the Secretome

- Systemic and Tissue Alterations: Injured pulmonary endothelial cells (ECs) actively drive pulmonary hypertension (PH) pathogenesis. Rather than acting as a passive barrier, the dysfunctional endothelium alters the secretion of multiple local factors that regulate smooth muscle cell differentiation, local coagulation cascade activation, perivascular cell migration, and angioproliferative remodeling [6,70].
- The Transpulmonary Gradient Advantage: While tissue studies from explanted lungs reveal altered endothelial gene expression, they fail to track the real-time secretome of a living patient. Measuring the dynamic transpulmonary content of endothelial-derived proteins bypasses systemic dilution, directly capturing active endothelial activation and vascular shedding under mechanical stress.
- Candidate Targets: Key molecular candidates include endothelin-1, vascular endothelial growth factor (VEGF) driving aberrant EC proliferation [71], the prostacyclin/thromboxane ratio, the MIF/CD74 signaling complex [72], and tumor necrosis factor-related apoptosis-inducing ligand (TRAIL), which directly mediates smooth muscle cell migration and vascular proliferation [73].

### 5.2. Perivascular and Circulating Inflammation

- Systemic and Tissue Alterations: Perivascular inflammation is a primary driver of vascular remodeling, with adventitial inflammation preceding intimal and medial layer involvement. Histological lesions exhibit distinct endophenotypes: the adventitia demonstrates complement activation and accumulation of inflammatory cells (dendritic cells, fibroblasts, macrophages, and lymphocytes), while plexiform lesions express prominent interferon and tumor necrosis factor-alpha (IFN/TNF-α) signatures [64,74,75]. High-throughput peripheral proteomics find elevated circulating levels of chemokines in high-risk idiopathic PAH (iPAH) patients [76].
- The Transpulmonary Gradient Advantage: While systemic blood-based signatures support dysregulated immunity, they lack organ specificity and do not fully resolve local pulmonary pathophysiology from background systemic inflammation [7]. Transpulmonary sampling during exercise aims to isolate local pulmonary inflammatory secretion from systemic immune noise.
- Candidate Targets: Direct transpulmonary evidence for inflammatory mediators is limited and differs between conditions. In heart failure with reduced ejection fraction (HFrEF) and high PVR, net pulmonary release of IL-6, IL-33, CCL4, MCP-3 (CCL7) and CXCL10 was reported, together with pulmonary uptake of oncostatin M, CCL19 and CCL28; these gradients were negligible in controls and in patients with low PVR [60]. In contrast, in PAH, serum IL-6 was elevated in both arterial and mixed venous blood but showed no step-up across the lungs, suggesting that systemic IL-6 is not primarily determined by pulmonary release in that setting [77]. IL-1β, CCL5 and TNF-β remain candidates nominated by peripheral proteomics and tissue studies, without transpulmonary gradient data in PAH to date [76,78,79].

### 5.3. Metabolic and Lipidomic Remodeling

- Systemic and Tissue Alterations: PAH involves a metabolic shift toward uncoupled glycolysis (a Warburg-like phenotype) within both the remodeling pulmonary vascular wall and the struggling right RV. Systemic metabolomic profiling has linked distinct circulating metabolite alterations to severe RV dysfunction [79] and an increased risk of death [77].
- The Transpulmonary Gradient Advantage: Physical exertion amplifies active metabolites across the cardiopulmonary circuit. Capturing stage-specific metabolite gradients during exercise can possibly unmask net uptake or release of metabolic substrates by the lungs and RV under stress, which may not be evident at rest.
- Candidate Targets: Transpulmonary evidence is more developed for metabolites than for most protein classes but is largely restricted to resting conditions. In a dyspnea cohort stratified by resting PVR, pulmonary release of IDO-pathway tryptophan metabolites (kynurenine, anthranilate, quinolinate) was observed only in patients with PVR > 2 WU [77]. In connective tissue disease–associated pulmonary vascular disease, simultaneous pulmonary and radial arterial sampling showed exercise-stage-specific gradients, including net lactate uptake at peak exercise in patients with more advanced disease and implicated acylcarnitines, glycolytic intermediates and tryptophan catabolites [80]. Resting trans-right-ventricular and transpulmonary gradients of acylcarnitines, dicarboxylic acids and other lipid species have also been described in pediatric PAH [69]. Pulmonary arterial sampling at rest and peak exercise indicated that the metabolic profile of exercise PH lies between that of controls and PAH [68], although this single-site design cannot resolve organ-level exchange. Among lipid mediators, 22% of the eicosanoids profiled in a dyspnea cohort that excluded PAH showed a significant resting transpulmonary gradient, mostly uptake, and PGG2 uptake was associated with spirometry obstruction and COPD rather than with PH [78]; its relevance to PAH and to exercise therefore remains hypothetical. TCA cycle intermediates and amino acids remain candidates nominated by systemic profiling [77,79].

### 5.4. Extracellular Matrix Remodeling

- Systemic and Tissue Alterations: The progression of obliterative vascular lesions requires the constant degradation and deposition of the vascular extracellular matrix (ECM). Endostatin, a collagen-derived fragment reflecting matrix turnover, is highly elevated in systemic circulation, directly correlates with disease severity, and predicts poor clinical outcomes in PAH patients [81].
- The Transpulmonary Gradient Advantage: Single-site peripheral measurements cannot distinguish whether circulating matrix markers originate from systemic vascular beds, hepatic clearance, or the pulmonary circuit itself. Gradient analysis restricts the area of analysis of detectable changes in matrix marker concentrations, allowing for a more localized evaluation of the vasculopathy dysfunction between the sampling sites.
- Candidate Targets: Key proteomic targets include matrix metalloproteinases (MMPs), tissue inhibitors of metalloproteinases (TIMPs), and circulating fragments of collagen degradation such as endostatin. Gradient evidence is limited: in HFrEF, MMP9 was taken up across the lungs, with greater uptake at high PVR, and uptake of the extracellular matrix protein prolargin (PRELP) was observed only in the high-PVR group [60]. Endostatin, other MMPs and TIMPs have been characterized in PAH only in peripheral blood [81] and have not yet been tested by transpulmonary sampling.

### 5.5. Neurohormonal Axes and Cardiopulmonary Stress

- Systemic and Tissue Alterations: Autonomic nervous system dysregulation and persistent sympathetic overdrive lead to the progressive downregulation of myocardial *β*-adrenoreceptors, depletion of tissue norepinephrine, and impaired adenylate cyclase stimulation, driving right ventricular remodeling and changes in myosin heavy chain isoforms [82,83].
- The Transpulmonary Gradient Advantage: Exercise RHC provides a simultaneous snapshot of homeostatic, multisystem interactions. Measuring transpulmonary neurohormonal gradients during peak exercise could generate hypothesis about mechanical and neurohormonal strain coupling between the right ventricle and the pulmonary vasculature, with the caution that intrapulmonary sampling does not itself measure RV exchange,
- Candidate Targets: Critical candidates include brain natriuretic peptide (BNP), N-terminal pro-BNP (NT-proBNP), and adrenomedullin. Pulmonary BNP uptake has been demonstrated: in HFrEF with high PVR, BNP was taken up across the lungs, a finding interpreted as enhanced counter-regulatory cGMP signaling. NT-proBNP and adrenomedullin were elevated in pulmonary arterial blood of HFrEF patients versus controls but did not show significant transpulmonary gradients in that study [60]. That study was performed at rest, in decongested patients, and did not address exercise or ventricle–arterial coupling. Since neurohormonal activation is a shared feature of RV stress across PH etiologies, these mechanisms plausibly extend to PAH as well [84,85].

**Figure 3:**
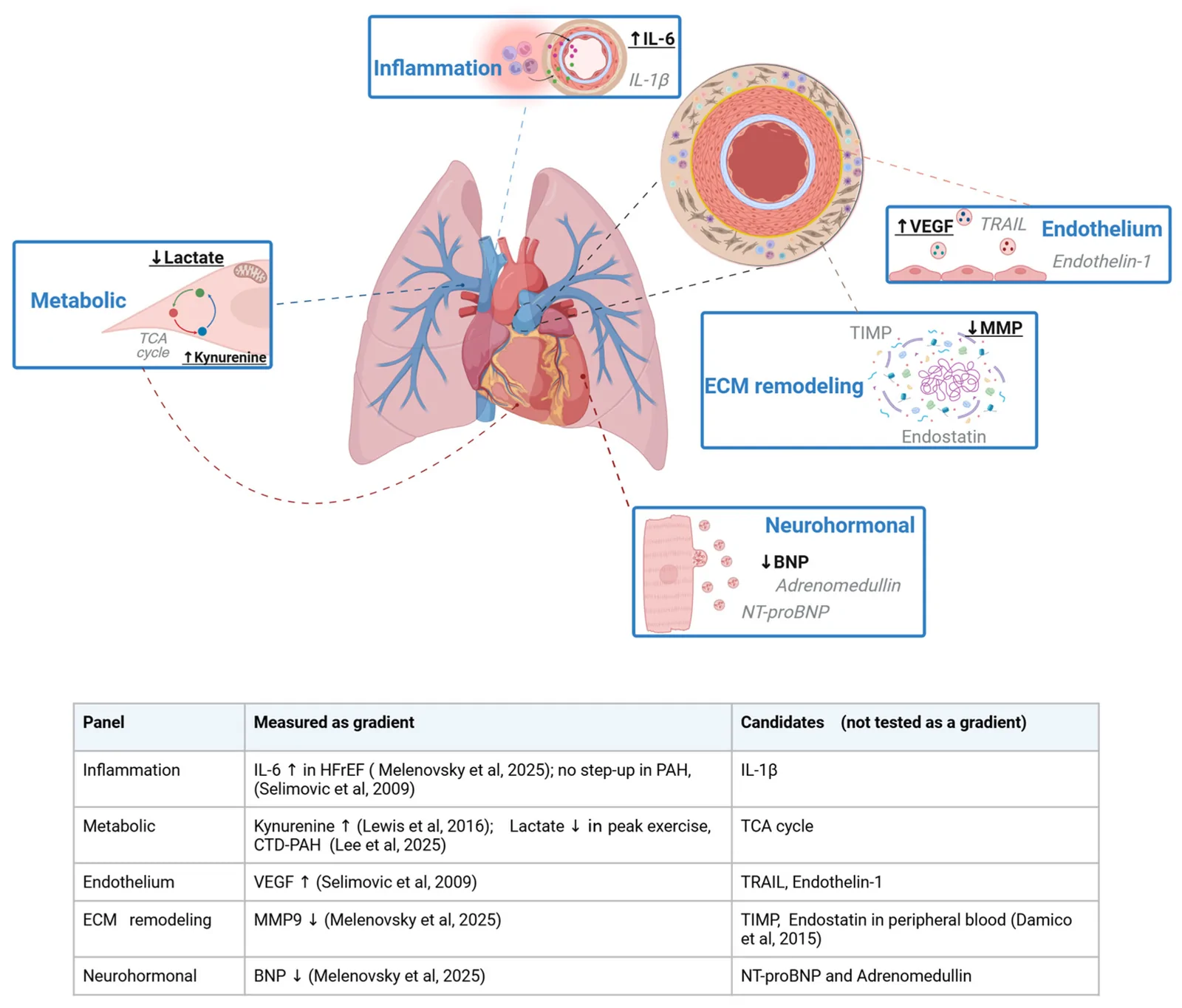
Biological domains in which exercise transpulmonary profiling is proposed to add information and classification of measured molecules in transpulmonary gradients and possible candidates [60,61,77,80,81]. Analytes in bold with arrows have a measured transpulmonary gradient in at least one human cohort (↑ net release, ↓ net uptake; the references and clinical context are detailed in the table included in the figure). Analytes in gray are candidates nominated by tissue or peripheral blood studies and have not yet been tested by transpulmonary sampling. Most gradients were measured at rest or in heart failure; only lactate was measured during exercise [80]. The figure does not imply that these measurements are validated for screening, monitoring or treatment selection. Molecular pathways of pulmonary vascular remodeling and metabolic shifts accessible via transpulmonary gradient profiling during exercise right heart catheterization. ECM: extracellular matrix. MMP: matrix metalloproteinase. TIMP: tissue inhibitors of metalloproteinases. TCA: tricarboxylic acid. VEGF: vascular endothelial growth factor. TRAIL: tumor necrosis factor-related apoptosis-inducing ligand. BNP: brain natriuretic peptide. Created in BioRender. Menezes, T. (2026) https://BioRender.com/ljh6ujx.

Together, these observations (Figure 3, Table 2) support exercise-induced transpulmonary profiling as a physiological assay of net pulmonary exchange, capable of linking hemodynamic stress to molecular mechanisms and disease heterogeneity in PH.

## 6. From Mechanistic Discovery to Endotype-Based Precision Medicine

Current standard initial therapies target pulmonary vasoconstriction and are primarily validated in patients with already advanced disease, historically characterized in clinical trials by a resting RHC with a PVR > 3 WU and mPAP > 25 mmhg [86]. Despite major advances, early disease remains difficult to detect using standard resting parameters [87]. Incorporating exercise hemodynamics improves diagnostic sensitivity in early PH. When paired with transpulmonary proteomics, this provocative strategy may detect subtle endothelial dysfunction and localized inflammatory shedding before rest RHC. A proposed application for this sensitive molecular window is interesting for screening asymptomatic, high-risk populations, such as BMPR2 mutation carriers and patients with systemic sclerosis, where it is still unknown whether early intervention could potentially alter the disease trajectory.

We are entering an era of precision medicine where identifying biomarkers and disease phenotypes is essential to improving mechanistic studies and clinical care [17]. Pulmonary hypertension is heterogeneous, not only in its clinical presentation but also in its underlying molecular architecture. By applying advanced omics clustering and machine learning algorithms to exercise-induced transpulmonary gradients, it may become possible to define biologically coherent subgroups or “endotypes.” These molecular subgroups cut directly across traditional, rigid World Health Organization (WHO) clinical classifications. Mapping these distinct patient clusters could allow for more precise therapeutic targeting, ensuring that individual patients receive interventions tailored to their specific active pathological pathways.

Because RHC is already a standard diagnostic and follow-up test in PH management, tracking transpulmonary protein content in principle could be a way to monitor treatment response throughout disease progression [80]. Evaluating transpulmonary gradients at baseline and following targeted therapy could help indicate whether a drug is modifying the pulmonary vascular molecular microenvironment, though this longitudinal application has not yet been tested. Such a signal would be more proximal and organ-specific than traditional distal clinical markers like the 6-minute walk distance (6 MWD) or WHO functional class, a potential advantage that remains to be established through direct comparative sensitivity data. Furthermore, this framework may nominate candidate targets for therapeutic discovery: molecules that demonstrate consistent, net transpulmonary secretion into the systemic circulation during disease—but remain neutral or extracted in healthy cohorts—represent candidate targets warranting formal validation in future mechanistic and pharmacological studies.

Although RHC is an invasive procedure, it is often performed in an outpatient setting. In a multicenter study of RHC procedures performed in patients with PH (including vasoreactivity testing and pulmonary angiography), procedure-related morbidity was 1.1% and mortality 0.055%. Most common complications were related to vascular access, such as hematoma at the puncture site [38,88]. This estimate refers to resting procedures and does not capture exercise, radial arterial cannulation, or repeated dual-site sampling; the incremental risk of these additions has not been systematically quantified, and exercise RHC has been reported as feasible in expert centers [35]. Practical limitations of the procedure include access to referral centers, differences in load-increase protocols, patient positions, complex physiologic interpretation, and technical demands for simultaneous dual-site sampling during exercise [32]. Also, high-throughput omics arrays remain costly with large-scale validation across diverse PH phenotypes still limited [7]. Finally, the transpulmonary gradient is a net measure of lung transit and cannot resolve spatial heterogeneity within the organ [58].

The combination of exercise stress testing and transpulmonary proteomic profiling transform a standard hemodynamic assessment into a dynamic, functional molecular biopsy [35]. To successfully transition this approach from a mechanistic discovery tool into routine precision medicine, large-scale validation across diverse clinical populations is mandatory. There is an urgent need to establish multi-center collaborative networks and standardized sample-processing protocols. Only through these unified platforms can we validate, scale, and fully harness exercise-induced transpulmonary gradients to guide target-directed therapies and reshape the future of pulmonary vascular medicine.

## 7. Limitations of the Current Evidence Base

Beyond the procedural constraints above, the evidentiary foundation for this narrative review remains preliminary and warrants explicit acknowledgment. With few exceptions, most of the available evidence comes from small and single-center studies without longitudinal monitoring. Therefore, the treatment-monitoring and target-discovery applications proposed in this review are hypothesis-generating extrapolations rather than demonstrated capabilities. The strongest large-cohort support for the method’s feasibility [60] comes from a Group 2 PH-HFrEF population with a distinct primary pathobiology from the Group 1 PAH mechanisms. This evidence should be interpreted as validating the transpulmonary sampling method itself in a cardiopulmonary population at rest, rather than validating the PAH-specific molecular candidates discussed here. Finally, proteomic platform choice materially affects which gradients are detectable, and known modest inter-platform concordance between current affinity-based technologies means that single-platform findings should be interpreted as platform-specific until cross-platform replication is available.

## 8. Conclusions

The integration of exercise right heart catheterization with multi-omics transpulmonary gradient analysis offers a promising paradigm for evaluating pulmonary vascular disease. By capturing real-time molecular concentration under physiological stress, this functional molecular biopsy has the potential to bypass the confounding effects of systemic blood dilution and the clinical risks of tissue biopsy. The resulting dynamic gradients offer an objective metric of net pulmonary secretion and clearance, providing a window into early endothelial activation, perivascular inflammation, and metabolic shifts that remain largely hidden at rest.

Within the broader landscape of precision medicine, this methodology addresses a critical gap in pulmonary hypertension. Current clinical frameworks rely heavily on macro-vascular hemodynamics and distal clinical metrics that only register abnormalities once significant, irreversible structural remodeling has occurred. Transitioning from peripheral, single-site biomarker sampling to organ-specific, stress-induced transpulmonary gradients allows the field to move past descriptive clinical phenotypes and toward biologically coherent molecular endotypes. This shifts the diagnostic and therapeutic focus toward localized, proximal mechanisms of disease.

Realizing this potential requires two concrete steps beyond the standardization already discussed: explicit, published protocols for exercise load escalation and cross-platform validation between affinity-based proteomic technologies, deployed within large-scale, prospective longitudinal cohorts spanning at-risk individuals and distinct clinical phenotypes. Only this combination can bind specific transpulmonary molecular signatures to long-term clinical outcomes and treatment response, moving this framework from proof-of-concept toward target-directed precision medicine in pulmonary hypertension.

## Figures and Tables

**Table 1:** Existing approaches to PH workup and its characteristics.

Approach	Procedural Invasiveness	Repeatable and Sensitive to Disease Progression *	Lung-Specific	Early-Stage Sensitivity for Diagnosis
Resting RHC/ biomarkers	Low	Yes	No	No
Tissue biopsy	High	No	Yes	Limited
Animal models	N/A	Limited	Yes	Yes
Precision-cut lung slices (PCLS)	High (ex vivo)	No	Yes	Limited
Exercise RHC + transpulmonary omics	Moderate	Yes	Partial	Yes

* Refers to the feasibility and safety of performing repeated tests to establish worsening in vascular remodeling and cardiac function. These entries denote proposed rather than demonstrated capabilities.

**Table 2:** Key primary studies reporting transpulmonary gradients, or exercise sampling, in pulmonary vascular disease.

Ref.	Study (Design)	Population	N Analyzed	Sampling Sites	Rest/Exercise Conditions	Molecular Class	Principal Finding
[61]	Selimovic et al., 2009 (prospective, cross-sectional)	PAH (IPAH 16, CTD-PAH 24, other 4; 43 PAH plus 1 peripheral pulmonary stenosis) vs. left-sided WPW controls without cardiac disease	44 PAH; 20 controls	PAH: radial artery and PA, simultaneous. Controls: PA and left atrium or femoral artery	Rest, 10 min after hemodynamic measurements	Serum proteins (ELISA): VEGF, PDGF-BB, TGF-β1, IL-6	Step-up of VEGF, PDGF-BB and TGF-β1 across the lungs in PAH, none in controls. No IL-6 step-up in either group; arterial IL-6 predicted mortality (HR 1.08). Gradients did not correlate with severity
[77]	Lewis et al., 2016 (derivation and validation cohorts)	Dyspnea referred for invasive CPET (MGH; LVEF ≥ 0.45; lung mechanical limitation excluded); validation cohorts including established PAH	Derivation 71; validation 71; PAH vs. controls 11 vs. 19. N for gradient analysis not stated	Radial artery vs. proximal PA	Gradient stratified by resting PVR (≤2 vs. >2 WU); circulating levels correlated with exercise PVR and ΔPQ	Plasma metabolites (LC–MS): IDO tryptophan metabolites	Pulmonary release of kynurenine, anthranilate and quinolinate (radial > PA, *p* < 0.05) only with PVR > 2 WU; no gradient with normal PVR
[68]	Sanders et al., 2019 (matched cohort)	Controls with normal exercise hemodynamics, exercise PH, resting PAH	26 per group	PA only (no gradient)	Rest and peak exercise	Plasma metabolites (unbiased MS)	Exercise-PH signature lies between controls and PAH; paired rest–exercise changes classified groups with 88–96% accuracy. Not a gradient study
[69]	Chouvarine et al., 2020 (prospective)	Paediatric PAH without shunt vs. non-PAH controls, clinically indicated catheterization	Metabolomics 8 PAH + 8 controls; lipidomics 8 PAH + 9 controls; per-metabolite n reduced by missing-value exclusion	SVC, PA, ascending aorta (transpulmonary = ascending aorta vs. PA)	Rest	Plasma metabolites and lipids (Metabolon, Lipidyzer)	7 metabolites with differential transpulmonary gradients in PAH vs. controls (FDR < 0.15), e.g., N6-acetyllysine step-up, azelate step-down; correlated with hemodynamic and echocardiographic variables. Trans-RV gradients dominated by lipids (octadecanedioate, stearoylcarnitine)
[80]	Lee et al., 2025 (cross-sectional)	Connective tissue disease with established or suspected PAH	63 patients; 57, 53, 56, 56 plasma samples (baseline, free-wheel, peak, recovery)	PA and radial artery, simultaneous	Baseline, free-wheeling, peak exercise, recovery	Plasma metabolites (UHPLC–MS)	Gradients were exercise-stage specific and correlated with hemodynamics mainly during free-wheeling. Net lactate uptake at peak exercise in more advanced disease; acylcarnitines, glycolytic intermediates and tryptophan catabolites relevant
[60]	Melenovsky et al., 2025 (cross-sectional)	HFrEF (LVEF ≤ 40%), decongested, vs. controls without HF	21 controls; 40 HF low PVR; 40 HF high PVR (from 160 HFrEF)	Wedged (O2 sat > 90%) vs. unwedged PA; gradient = wedged − PA	Rest	Plasma proteins (Olink PEA; Inflammation, CVII, CVIII panels)	High PVR: 23 significant gradients (18 uptake, 5 release). Uptake of OSM, MMP9, CCL19, BNP, GDF2/BMP9; release of IL-6, IL-33, CCL4, MCP-3, CXCL10. Low PVR: 5; controls: 1 (CCL19)
[78]	Mounsey et al., 2026 (cross-sectional)	Dyspnea referred for spirometry and RHC; history of PAH excluded	516	PA and radial artery	Rest	Plasma eicosanoids and oxylipins (LC–MS)	194 of 887 metabolites with significant gradients (74% uptake); less PGG2 uptake associated with higher FEV1/FVC and lower odds of obstruction and COPD

## Data Availability

No new data were created or analyzed in this study. Data sharing is not applicable to this article.
